# Muscle Fiber Viability, a Novel Method for the Fast Detection of Ischemic Muscle Injury in Rats

**DOI:** 10.1371/journal.pone.0084783

**Published:** 2014-01-13

**Authors:** Zsolt Turóczi, Péter Arányi, Ákos Lukáts, Dávid Garbaisz, Gábor Lotz, László Harsányi, Attila Szijártó

**Affiliations:** 1 1^st^ Department of Surgery, Semmelweis University, Budapest, Hungary; 2 Department of Human Morphology and Developmental Biology, Semmelweis University, Budapest, Hungary; 3 2^nd^ Department of Pathology, Semmelweis University, Budapest, Hungary; University of Tübingen, Germany

## Abstract

Acute lower extremity ischemia is a limb- and life-threatening clinical problem. Rapid detection of the degree of injury is crucial, however at present there are no exact diagnostic tests available to achieve this purpose. Our goal was to examine a novel technique - which has the potential to accurately assess the degree of ischemic muscle injury within a short period of time - in a clinically relevant rodent model. Male Wistar rats were exposed to 4, 6, 8 and 9 hours of bilateral lower limb ischemia induced by the occlusion of the infrarenal aorta. Additional animals underwent 8 and 9 hours of ischemia followed by 2 hours of reperfusion to examine the effects of revascularization. Muscle samples were collected from the left anterior tibial muscle for viability assessment. The degree of muscle damage (muscle fiber viability) was assessed by morphometric evaluation of NADH-tetrazolium reductase reaction on frozen sections. Right hind limbs were perfusion-fixed with paraformaldehyde and glutaraldehyde for light and electron microscopic examinations. Muscle fiber viability decreased progressively over the time of ischemia, with significant differences found between the consecutive times. High correlation was detected between the length of ischemia and the values of muscle fiber viability. After reperfusion, viability showed significant reduction in the 8-hour-ischemia and 2-hour-reperfusion group compared to the 8-hour-ischemia-only group, and decreased further after 9 hours of ischemia and 2 hours of reperfusion. Light- and electron microscopic findings correlated strongly with the values of muscle fiber viability: lesser viability values represented higher degree of ultrastructural injury while similar viability results corresponded to similar morphological injury. Muscle fiber viability was capable of accurately determining the degree of muscle injury in our rat model. Our method might therefore be useful in clinical settings in the diagnostics of acute ischemic muscle injury.

## Introduction

Acute long-lasting arterial occlusions represent serious clinical problems due to their frequent occurrence (incidence: 15/100000) and severe complications [1]. Even recent studies put post-operative limb loss between 10 and 30%, and post-operative mortality between 10–20% [1]–, both of which are direct consequences of the severe ischemic-reperfusion injury to the extremities. Prompt and proper diagnosis is therefore important. The Rutherford classification is widely used for staging (Stages I–III) the severity of acute ischemic injury of the limbs in the clinical practice [4], designed to determine the urgency of a revascularization procedure [5]. Assessment of the degree of ischemic injury within a clinically relevant time-frame however still remains unsolved.

Rapid determination of the precise degree of ischemic injury is of great clinical importance [6], whereas revascularization of a severely injured extremity might aggravate complication rates and mortality. In case of irreversible injury amputation is the only solution to avoid serious life threatening complications [7].

The aim of the current study was to describe and evaluate a technique – muscle fiber viability measurements on frozen sections of muscle biopsies – which has the capability to assess the degree of ischemic injury in a short period of time. Comparing the viability results with the morphological evaluations of muscle injury, our study shows that this technique is a reliable detection tool which, if adapted to clinical practice, could undoubtedly help to determine the severity of muscle damage, therefore to facilitate therapeutic decisions.

## Materials and Methods

### Animals and ethics statement

Male Wistar rats (n = 42) weighing 220–250 grams were used (Charles Rivers Hungary Ltd, Budapest, Hungary). The experimental design was carried out in strict accordance with the recommendations in the Guide for the Care and Use of Laboratory Animals of the National Institutes of Health. The protocol was approved by the Committee on Animal Experimentation of Semmelweis University (Permit Number: 22.1/794/003/2009). All surgeries were performed under general anesthesia, with efforts made to minimize suffering. The animals were kept under specific, pathogen-free conditions in 12-hour day-night cycles at 22–24°C with unlimited access to commercial pellets and water. Each experiment was started at the same time of day to avoid the effects of circadian rhythm.

### Experimental design

Under general anesthesia the right jugular vein was cannulated for administration of anesthetics (ketamine and xylasine; 25 and 2.5 mg/bwkg/h respectively) and saline solution (3 mL/bwkg/h). Body temperature was maintained between 36.5 and 37.5°C by a heating pad connected to a rectal thermometer (Homoeothermic Blanket Control Unit, Harvard Apparatus, Holliston, MA).

The experiment was divided into two parts: in the first part the degree of ischemic injury alone was investigated with different exclusion times, while in the second part, the additive effect of reperfusion was studied using long-lasting exclusions followed by reperfusion. Muscle samples were collected for muscle fiber viability measurements and – to see if the data coincided with morphological signs of injury – also for light- and electron microscopic examinations.

#### Ischemic experiment

Through a median laparotomy the infrarenal section of the abdominal aorta was exposed. Four, 6, 8 and 9 hours of bilateral lower limb ischemia was established via infrarenal aortic occlusion [8]. After inducing ischemia, the abdominal wall was sutured in two layers. No reperfusion was allowed in this experiment. Samples were taken at the end of ischemia from the anterior tibial muscle.

#### Reperfusion experiment

8 and 9 hours of infrarenal occlusion were followed by 2 hours of reperfusion. Five minutes prior to reperfusion 60 IU Na-heparin was administered intravenously to mimic the clinical situation. After the specified time of ischemia the aortic occlusion was terminated and the abdominal wall was resutured in two layers. Samples from the anterior tibial muscle were harvested at the end of the reperfusion period.

Six additional animals were euthanized without any intervention to serve as untreated controls.

The experimental groups are summarized in Table 1.

**Table 1:** Experimental groups.

Group	Ischemic period (h)				Reperfusion (2 h)	n
	4	6	8	9		
Control	**-**	**-**	**-**	**-**	**-**	6
4I	**+**				**-**	6
6I		**+**			**-**	6
8I			**+**		**-**	6
9I				**+**	**-**	6
8IR			**+**		**+**	6
9IR				**+**	**+**	6

I: ischemia, IR: ischemia followed by reperfusion.

### Microcirculation

Laser Doppler Flowmeter (Moor DRT4, Moor Instruments Ltd, London, UK) was placed on the surface of the biceps femoris muscle to assess the alterations in microcirculatory flow during the ischemic period in all groups. The flow was evaluated as described by our team in detail previously [8].

### Viability assessment

Muscle samples collected from the left anterior tibial muscle were snap-frozen in liquid nitrogen and stored at −80°C until further processing. Three µm thick cross sections were made in a cryostat and stained for NADH-tetrazolium reductase (NADH-TR) enzyme-histochemical reaction [9]. Slides were incubated for 30 minutes at 37°C in a solution of nitroblue tetrazolium (1.8 mg/dL) and NADH (15 mg/dL) reagents (Sigma-Aldrich Inc, St. Louis, MO) in 0.05 M TRIS buffer (pH 7.6). Unused tetrazolium reagent was removed using ascending (30%, 60% and 90%) and then descending concentrations of acetone. Morphometric assessment of NADH-tetrazolium stained muscles was performed with an Olympus BX50 microscope equipped with Olympus DP70 high resolution camera (Olympus Corporation, Tokyo, Japan), using Leica QWin Pro (Leica Microsystems Ltd, Wetzlar, Germany) software (Color settings: red: 160/0, green: 130/0 and blue: 175/66 on RGB scale). Ten different fields were photographed randomly in each slide at 600× magnification. Viability of all fibers (total fiber viability) was calculated as a proportion of the total area of positive staining and the total area of muscle fibers in each picture. Furthermore, fibers were typed according to their staining characteristics with NADH-TR: lightly stained fibers were categorized as Type IIb (fast-twitch glycolytic), while fibers with intense staining were categorized as Type I (slow-twitch oxidative) [10], then the viability of each type was assessed separately with the software. Viability of a fiber type was calculated as a proportion of the total area of positive staining and total area of corresponding fibers (e.g. Type IIb or Type I) in each picture. The average of the 10 measurements was calculated for each animal. Final results are expressed as a percentage of the average of the untreated control muscles.

### Sample preparation for semithin sections and electron microscopy

After the desired time of ischemia or ischemia and reperfusion the right extremities were perfused through an intra-arterial catheter with warm (37°C) 4% paraformaldehyde in 0.1 M phosphate buffer (PB), followed by cold 2% glutaraldehyde solution (2% GA in 0.1 M PB) for a total of 30 minutes. Approximately 1×1 mm pieces of muscles were cut out and post-fixed in 2% glutaraldehyde (1 hour) followed by 1% osmium-tetroxide (in 0.1 M cacodylate buffer, 1 hour at 4°C). All pieces were dehydrated in graded alcohol series and embedded in araldite (Durcupan ACM Fluka, Sigma-Aldrich, St. Louis, MO). Ultrathin sections were prepared with an ultramicrotome, contrast-stained with uranyl acetate and lead citrate, and analyzed using a Hitachi H7500 transmission electron microscope (Hitachi Ltd, Tokyo, Japan). Electron micrographs were taken by an Olympus-SIS digital camera (Megaview II).

Semithin sections stained with toluidine blue were also prepared for *light microscopic* examination and inspected with a Zeiss Axiophot microscope equipped with AxioCamHRc digital camera (Carl Zeiss, Oberkochen, Germany).

The final montages from the pictures were prepared using Adobe Photoshop 7.0 (San Diego, CA) program.

### Statistical analysis

All values are expressed as means ±s.e.m. The assumption of normality was assessed with Shapiro-Wilk's test. Accordingly, one-way analysis of variance (ANOVA) was used for comparison of all groups in case of microcirculatory measurements, while in case of viability assessment two-way ANOVA was applied. Scheffe's post-hoc analysis was performed for between group comparisons. Data correlation was evaluated using Pearson's method. A 95% confidence interval was considered as statistically significant (P<.05). Statistical calculations were performed using IBM SPSS Statistics 20.0 software (IBM Corporation, Armonk, NY).

## Results

### Microcirculation

Microcirculatory flow was assessed throughout the ischemic period in all groups to evaluate whether this model is able to produce continuous ischemia without significant variations in flow. After occlusion, the flow dropped to 17.3±7.3% of the baseline flow and remained constant during the course of ischemia even 9 hours after the occlusion (16.5±9.2% of baseline). Flow values did not differ significantly in either of the ischemic groups, and remained below 30% in every animal, indicating that infrarenal aortic occlusion is capable of causing significant ischemia in the extremities with little or no individual variations and remaining constant throughout the whole experiment.

### Viability assessment – Ischemic experiment

**Figure 1:**
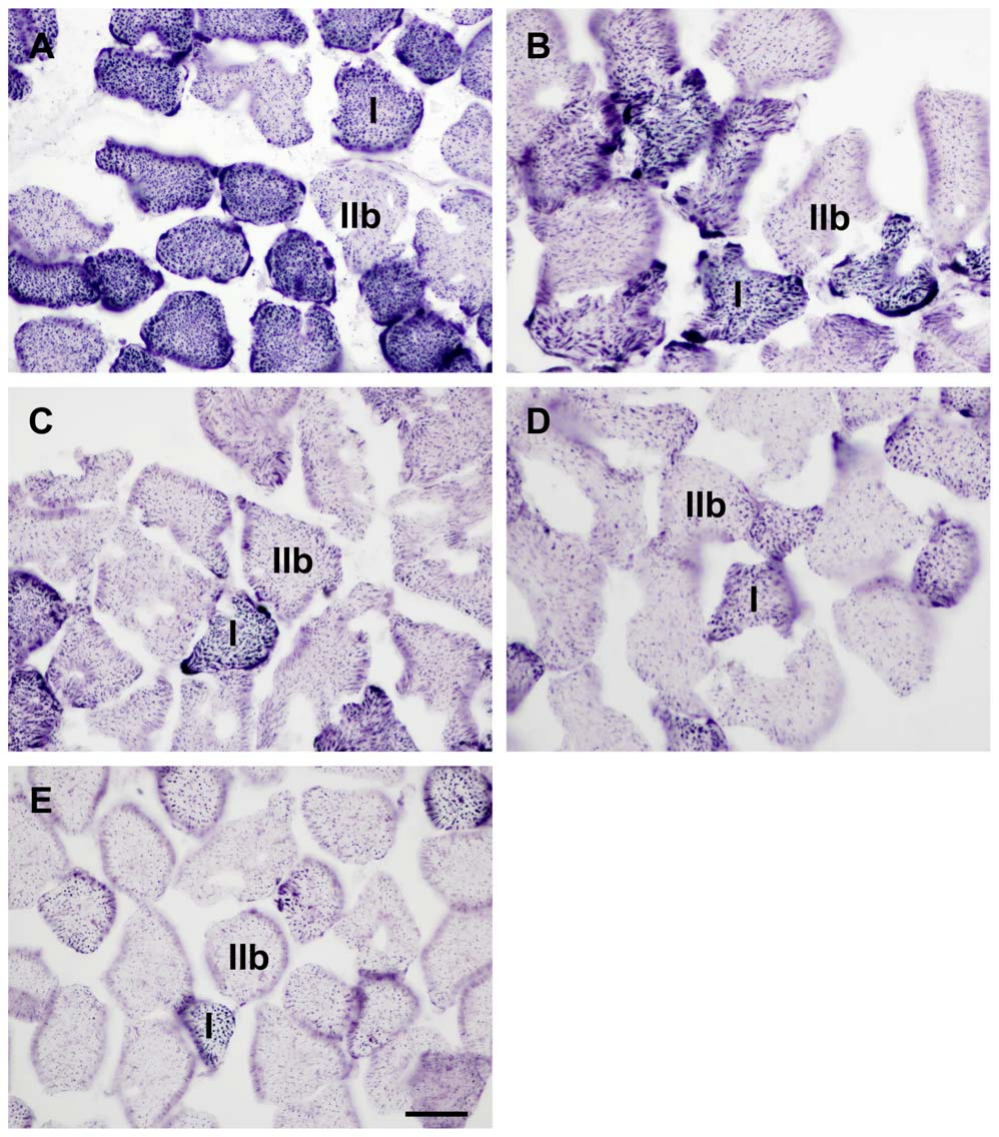
NADH-tetrazolium reductase stained sections from the ischemic experiment. The figure shows representative pictures taken from the anterior tibialis muscle of untreated control animals (A), as well as after 4 (B), 6 (C), 8 (D) and 9 hours (E) of ischemia (induced by infrarenal aortic occlusion) without reperfusion. Typical members of Type I and Type IIb fibers are marked on each picture as I or IIb, respectively. Bar: 35 µm.

**Figure 2:**
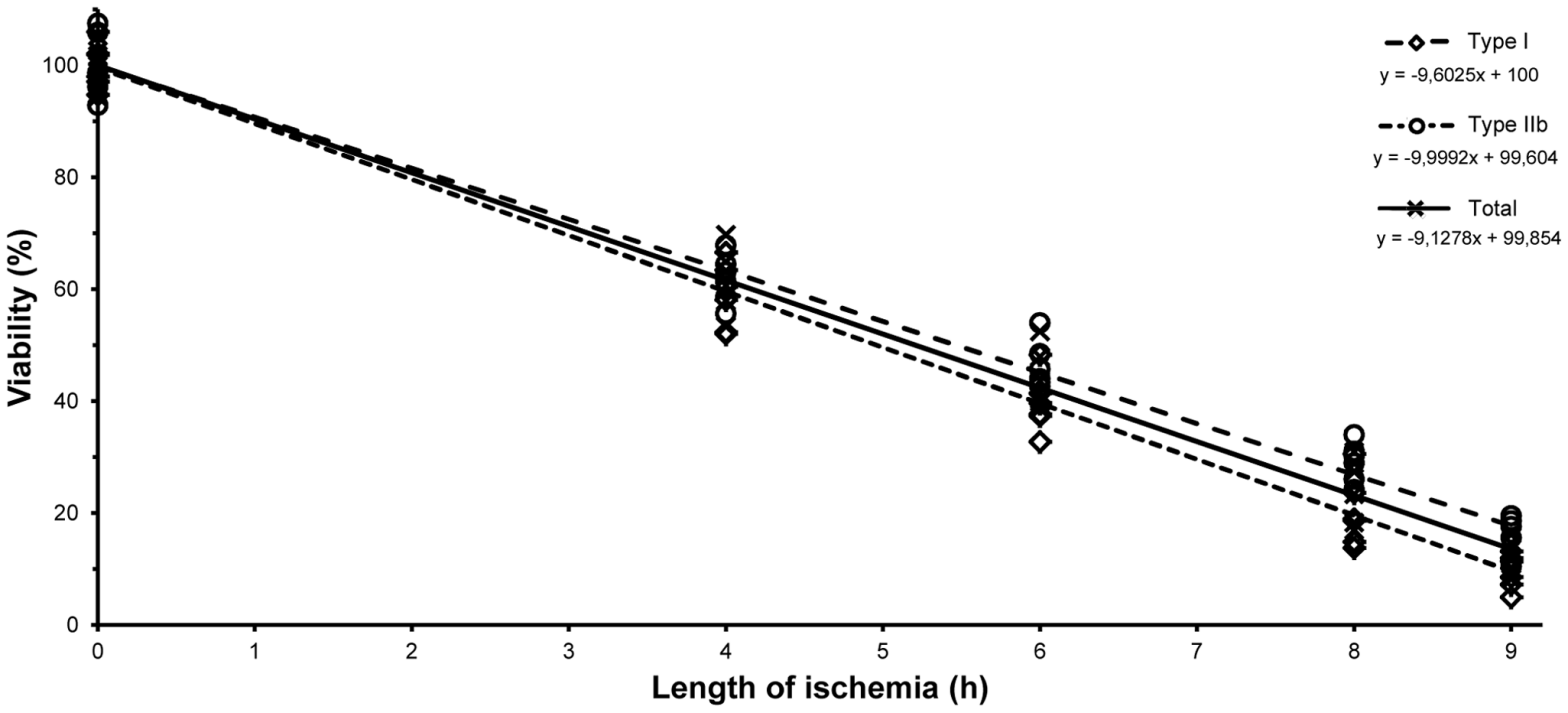
Changes in muscle fiber viability with the progression of ischemia regarding Type IIb, Type I fibers as well as total fiber viability. Muscle fiber viability decreased continuously over the time of ischemia, Pearson's analysis showed high correlation between the length of ischemia and muscle fiber viability in all three measurements. Type IIb fibers suffered greater damage compared to Type I fibers, which became significant after 6 hours of ischemia (P_6h_<.05, P_8h_<.01, P_9h_<.05). No significant differences (P>.05) could be found between total fiber viability and any fiber types (Two-way ANOVA with Scheffe's post-hoc correction). n = 6 per group.

Muscle viability as assessed by NADH-TR staining (Figure 1) decreased continuously over the time of ischemia with respect to all measurements (untreated control: Type IIb: 100.0±5.7%, Type I: 100.0±4.1%, total fiber viability: 100.0±3.9%; after 4 hours: 61.9±4.3%, 58.8±5.9%, 60.5±5.6%; 6 hours: 46.4±4.2%, 39.5±5.2%, 43.8±5.4%; 8 hours: 29.1±3.5%, 20.1±6.2%, 24.3±5.7%; 9 hours: 15.5±3.9%, 9.5±3.1%, 12.1±2.9%). Significant differences were found between the consecutive times of ischemia (P<.001) in all selectively assessed fiber types as well as in total fiber viability. Data analysis showed high correlation between length of ischemia and fiber viability in all measurements (Type IIb: R = −.989; R^2^ = .978; P<.001; Type I: R = −.990; R^2^ = .979; P<.001, total fiber viability: R = −.989; R^2^ = .978; P<.001) (Figure 2). Type I fibers suffered greater damage compared to Type IIb fibers, which became significant after 6 hours of ischemia (P_6h_<.05, P_8h_<.01, P_9h_<.05). No significant differences could be found between total fiber viability and any of the fiber types (P>.05).

### Light microscopy of semithin sections – Ischemic experiment

**Figure 3:**
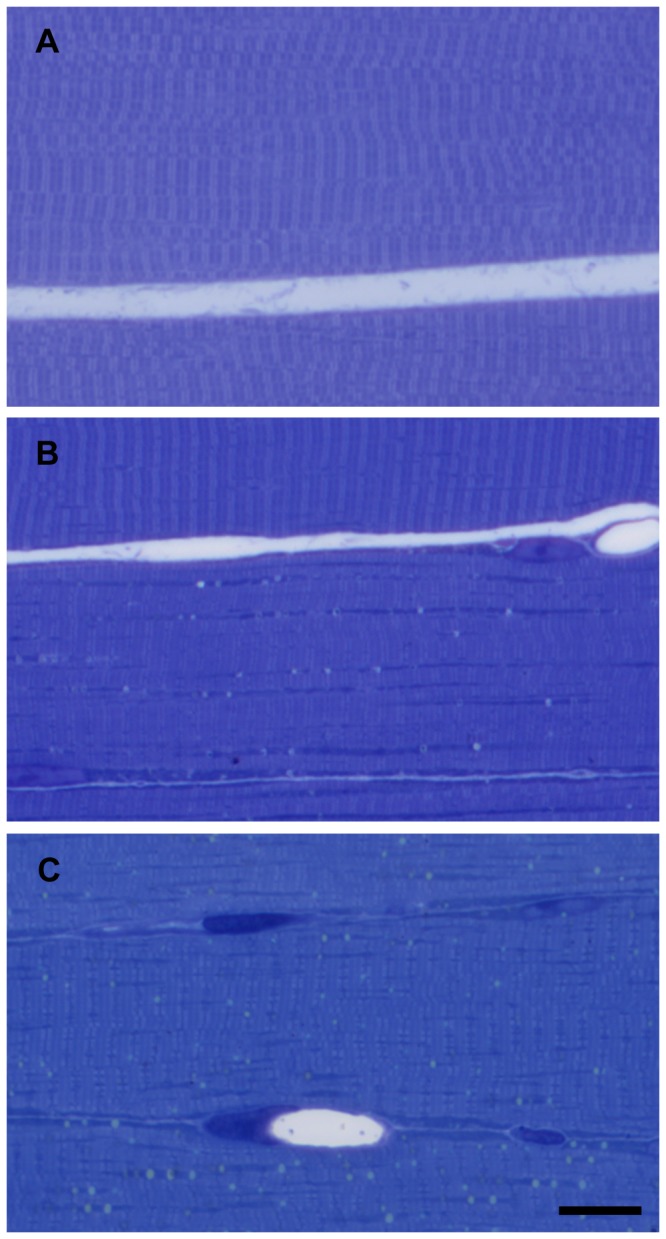
Morphology of muscle fibers in control animals and after 8 and 9 hours of ischemia. Normal morphology of muscle fibers, semithin sections from control muscle (A). After 8 hours of ischemia (B), mostly mitochondria-rich fibers seemed to be affected, while other fibers appeared to be normal. After 9 hours of ischemia (C), almost all fibers showed mild or moderate degree of damage. Toluidine blue staining. Bar: 20 µm.

In order to see if the decrease in muscle fiber viability coincides with the morphological signs of detectable injury, perfusion-fixed, semithin sections stained with toluidine blue were inspected. No detectable damage in muscle fibers of the untreated control animals was observed (Figure 3A). After 4 and 6 hours of ischemia no visible pathological changes were present either. Eight hours long ischemia (Figure 3B) resulted in slight hyperchromasia, with small homogeneous droplets of various densities appearing in some of the fibers. Mostly the fibers with higher mitochondria content - supposedly the red fibers - were affected. Nuclei showed signs of marginalization of heterochromatin. Capillary lumina seemed to be widened.

After 9 hours of ischemia hyperchromasia was more pronounced and a few hypercontracting fibers were detected. In some fibers cross striation became more pronounced, while becoming faded in others. In most of the fibers small homogeneous droplets of various densities were present, which were detectable even at low magnifications. At this stage, all fiber types contained these droplets, with emphasis on the mitochondria-rich fibers. The changes in nuclear morphology were more pronounced and widened capillaries were still present (Figure 3C).

### Electron microscopy – Ischemic experiment

**Figure 4:**
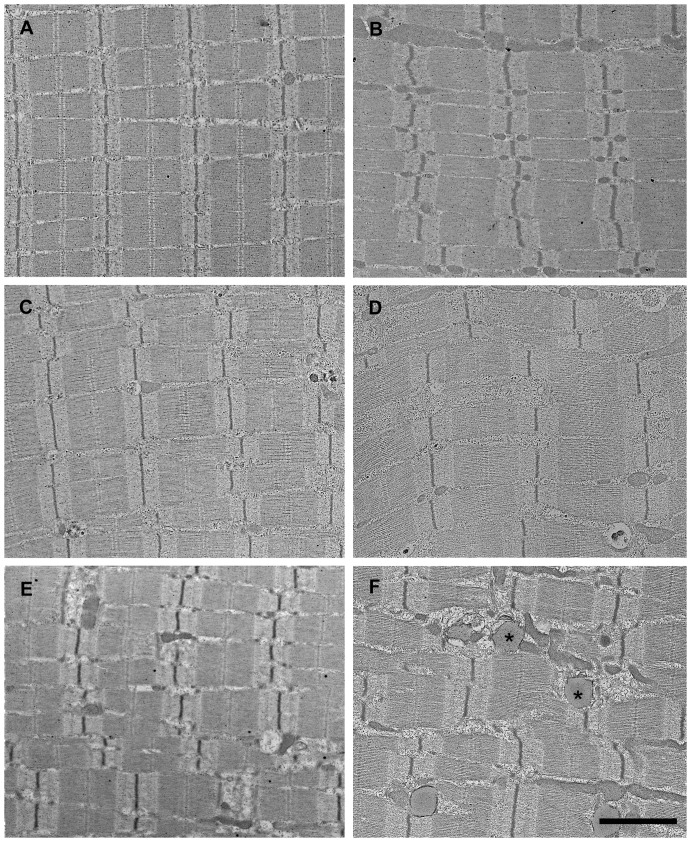
Ultrastructural changes in muscle fibers after 8 or 9 hours of ischemia. Fibers from control muscle displayed normal morphology both in mitochondria-poor (A) and mitochondria-rich muscle fibers (the latter were assumed to be red fibers) (B). After 8 (C, D) and 9 hours of ischemia (E, F) signs of morphological damages were already evident, several lipid-like homogeneous droplets with a peripheral zone of electron-dense margin could be seen (*), especially in mitochondria-rich fibers (D, F, right column). Bar: 2 µm.

In order to supply a more detailed description of the morphological signs of injury, electron microscopic appearance of the muscle fibers was studied. Electron micrographs of muscle fibers in control animals showed normal morphology and no appreciable damage (Figure 4A, B).

Eight-hours-long ischemia resulted in intact myofibrillar structures. Glycogen granules were markedly reduced and mitochondria were moderately swollen. Completely in line with the original places of mitochondria, lipid-like homogeneous droplets with a peripheral zone of electron-dense margin could be seen, primarily in mitochondria-rich muscle fibers. At the same locations, dense structures of various sizes could occasionally be observed (Figure 4C, D).

Nine hours of ischemia resulted in near complete loss of cellular glycogen content. The shape of mitochondria was variously deformed with occasional flocculent matrices. Moderate amounts of mitochondria showed signs of disruption, with myelin figures observable in some places. Lipid-like droplets similar to those observed in the 8I samples became more numerous. These lipid-like structures could also be found in the subsarcolemmal mitochondria clusters. The sarcoplasmic reticulum showed moderate degrees of vacuolization and disorganization. Segregation of euchromatic and heterochromatic components of the nucleus became more prominent. The observed changes appeared also in mitochondria-poor muscle fibers in a lower degree (Figure 4E, F).

### Viability assessment – Reperfusion experiment

**Figure 5:**
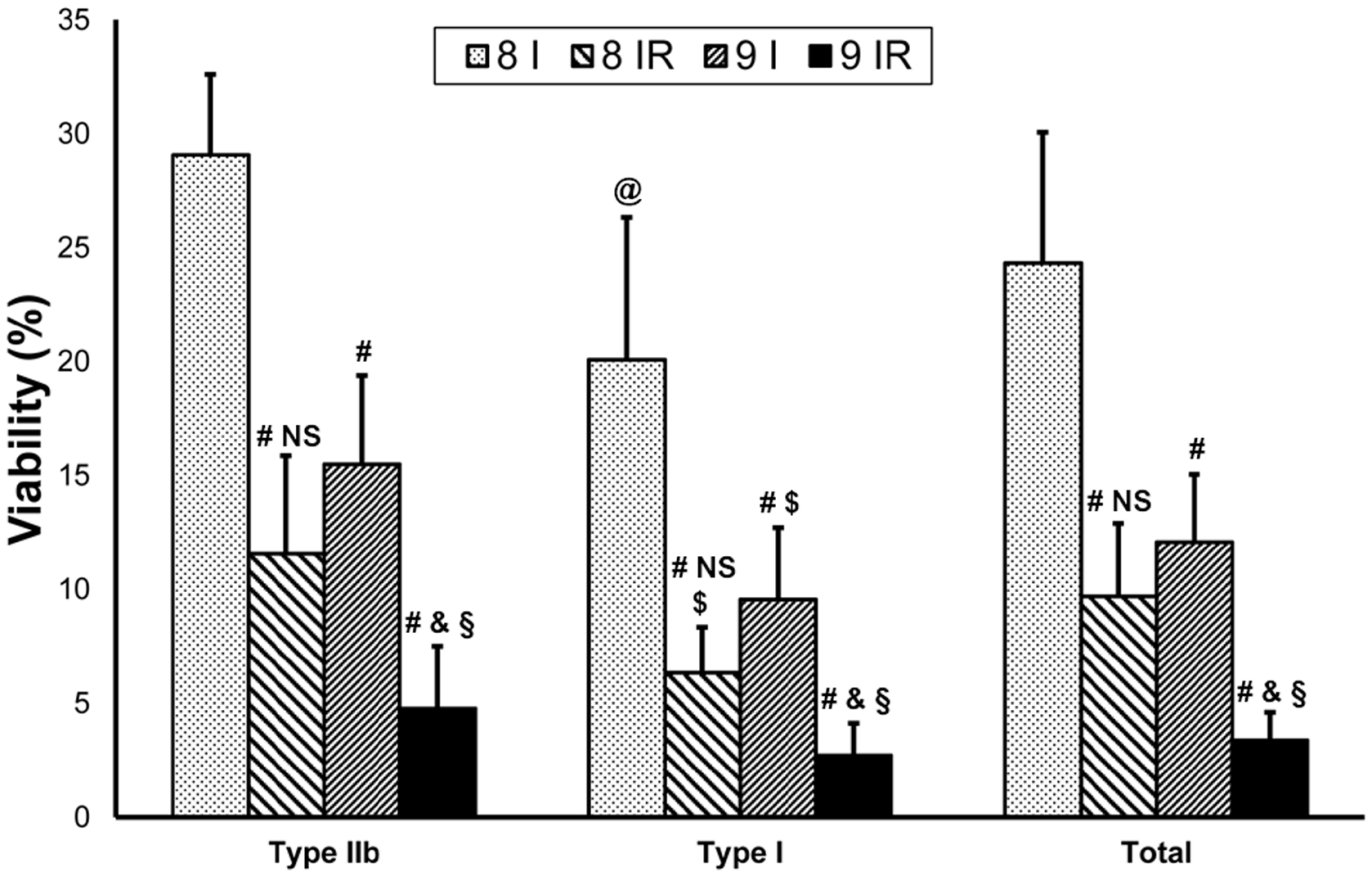
Viability results after long-lasting ischemia and reperfusion. Muscle fiber viability showed significant reduction after 8-hour-ischemia-only group regarding all three fiber measurements. Also, viability after 9 hours of ischemia and 2 hours of reperfusion decreased significantly compared to the 9-hour-ischemia-only group. Significant difference could be found between the two ischemia-only groups, nevertheless 8 hours ischemia followed by 2 hours reperfusion and 9 hours ischemia alone resulted in a similar loss of viability in all fiber measurements. Type I fibers showed significantly greater loss of viability compared to Type IIb fibers in the 8I, 8IR and 9I groups. Values are given as means ±s.e.m. Between group and within fiber comparison by Two-way ANOVA: P<.001; P<.001 respectively. Differences between the groups by Scheffe's post-hoc test were as follows: # P<.001 vs. corresponding 8I group; & P<.05 vs. corresponding 8IR group; § P<.001 vs. corresponding 9I group; NS P>.05 vs. corresponding 9I group. Differences within fibers by Scheffe's post-hoc test were: $ P<.05 vs. corresponding Type IIb fibers; @ P<.001 vs. corresponding Type IIb fibers; n = 6 per group.

Viability showed significant reduction (P<.001) in the 8IR group compared to the 8-hours-ischemia only (8I) group in both assessed fiber types, as well as in total fiber viability. Also, viability after 9 hours of ischemia and 2 hours of reperfusion (9IR) decreased significantly in all measurements compared to the 9-hours-ischemia group (P<.001). In the 8IR group Type I fibers showed significantly decreased viability (P<.05) compared to the Type IIb. No significant differences (P>.05) could be found between the fiber types in the 9IR group (Figure 5).

### Light microscopic evaluation of semithin sections – Reperfusion experiment

**Figure 6:**
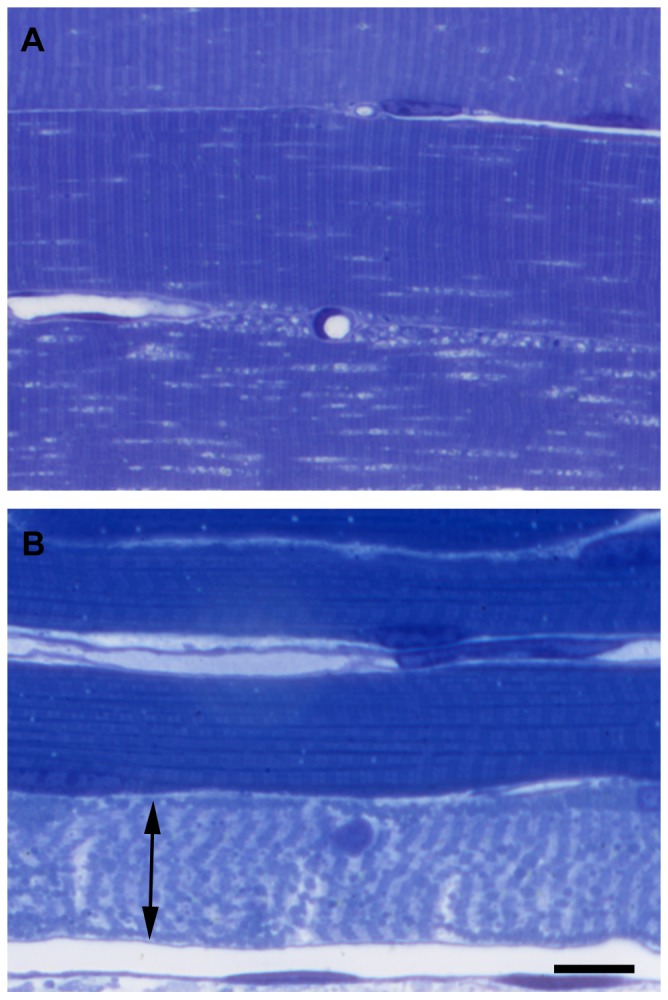
Morphological signs of ischemic-reperfusion damage of muscle fibers as demonstrated on semithin sections. Marked morphological changes were visible in all fibers even after 8(A), while after 9 hours of ischemia followed by 2 hours of reperfusion (B) necrosis (double arrow) was already evident in the majority of the fibers. Toluidine blue staining. Bar: 20 µm.

Eight hours of ischemia and 2 hours of reperfusion resulted in marked morphological changes in the semithin sections. In addition to the alterations visible in the eight-hour-ischemia-only group, a higher number of lipid-like droplets were observable in most of the fibers. In some muscle fibers thin clefts appeared between the myofibrils. Also, nuclei with marginal heterochromatin were frequent findings. As in other stages, mitochondria rich fibers showed higher degree of injury (Figure 6A).

Reperfusion after 9 hours of ischemia showed large numbers of necrotic fibers in semithin sections. Non-necrotic fibers showed the same morphological pattern of injury as in case of the 8IR group (Figure 6B).

### Electron microscopy – Reperfusion experiment

**Figure 7:**
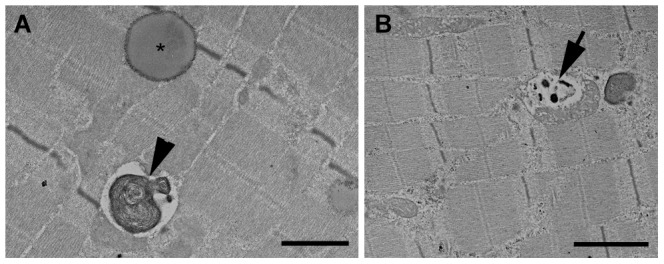
High magnification images of damaged muscle fibers from the 8IR group. Lipid-like droplets (*) and myelin figures (arrowheads) were frequently seen (A). Disrupted mitochondria with dense granules of various sizes appeared in the same position (B - arrow). Bar: 1 µm on figure A and 2 µm on figure B.

**Figure 8:**
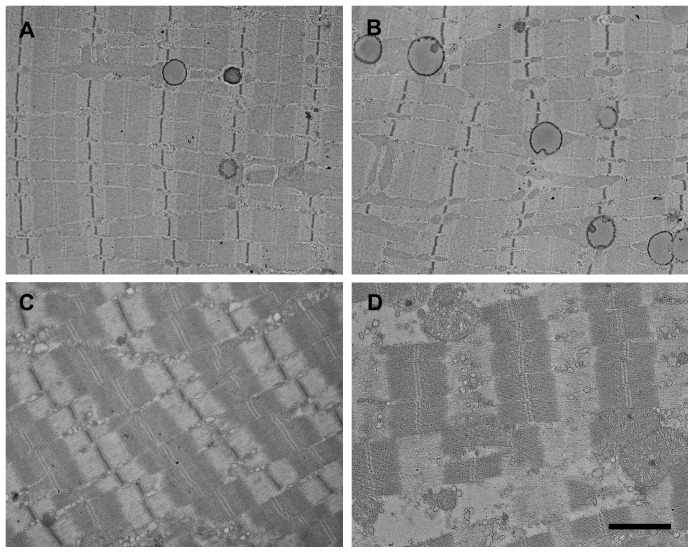
Reperfusion augments ultrastructural damage after 8 or 9 hours ischemia and 2 hours reperfusion. In the 8IR group, signs of degenerative changes similar to those observed in 8I group became more prominent on electron microscopic samples. Several lipid-like droplets were visible, being present in almost all fibers, both with lower (A) or higher amounts of mitochondria (B). Prominent swelling of the sarcoplasmic reticulum was also observed. In the 9IR group, several necrotic fibers were seen (D), while the surviving fibers belonged exclusively to the mitochondria-poor group (C). Bar: 2 µm.

Eight hours of ischemia followed by 2 hours of reperfusion caused absence of glycogen. Lipid-like droplets similar to those described previously were seen in extremely high numbers. Myelin figures were also frequently observed (Figure 7A), as was the disintegration of several mitochondria (Figure 7B). Prominent swelling of the sarcoplasmic reticulum was detectable. Similarly to other samples, pathological alterations were more prominent in mitochondria-rich fibers (Figure 8A, B).

Two hours of reperfusion after 9 hours of ischemia resulted in necrosis in the majority of fibers. Non-necrotic fibers showed similar but more pronounced alterations as in the 8IR group (Figure 8C, D).

## Discussion

Severe long-lasting acute lower limb vascular occlusions are associated with serious clinical conditions. The risk is high for the development of irreversible lesions with only one therapeutic option: amputation. However, bedside determination of the extent of muscle injury is not yet possible, thus irreversible injuries can not be separated in time from severe reversible ones.

In an attempt to resolve this problem our aim was to develop and evaluate a technique which could be suitable for rapid assessment of the degree of muscle injury, with the use of an experimental model mimicking clinical settings.

In most acute occlusive clinical situations significant residual flow is present due to the existence of collateral vessels [11], therefore ischemia is rarely complete [12]. Infrarenal aortic occlusion seems to be the most suitable method to mimic human acute occlusive situations in rats namely, during occlusion a certain degree of collateral flow is always present [13]. Even so, aortic exclusion is able to produce sufficient degree of ischemic injury [13], [14], therefore aortic occlusion was chosen for ischemia induction in the current study.

All animals were kept under anesthesia during the whole course of the experiment to eliminate fluctuations in flow caused by motion [13], [15]. Microcirculatory measurements were conducted to confirm that residual flow remained stable during the course of ischemia. In the current study, residual flow was constant throughout the whole ischemic period, making the results comparable and reproducible. Flow above 30% of the baseline was not detected in any individual rat, confirming that this type of occlusion was able to produce sufficient degree of ischemia [16].

Depletion of energy reserves results in cell death [17]. The mitochondria are the most important organella for energy transformation and since it has been demonstrated that mitochondrial function decreases during ischemia [18], detecting the dysfunction of these subcellular structures might be the most reliable method for identifying cellular injury [19]. Tetrazolium salt reduction is widely applied to indicate mitochondrial integrity [20] since decreased reaction implies organelle disruption [21]–[23], thus these salts are able to assess cellular viability.

Mostly spectrophotometric [24]–[29] and planimetric [30]–[35] methods are in general use for the quantitative assessment of tetrazolium reduction in vivo. Both methods are reported to correlate well with the extent of muscle injury [36], however these methods were mostly tested and proved as being effective after revascularization. Only limited data have been obtained so far about the use of planimetry under sole ischemic conditions [37] and in regard to spectrophotometry no data have been reported hitherto. Furthermore, these methods are unfortunately not suitable for bedside diagnostic purposes due to long preparation times and/or large sample requirements.

It was previously demonstrated that the intensity of NADH-TR staining on frozen sections decreases after a relatively long period of ischemia, even without reperfusion [38]. Whilst frozen sectioning is able to provide rapid sample preparation routinely utilized in various surgical conditions [39]–[41], the application of tetrazolium salt reduction on frozen sections could provide the basis for rapid determination of the degree of injury in case of acute limb ischemia. Regarding our new method the procession time mostly depends on the staining procedure, while quantitative assessment can be performed in few minutes. Namely, sampling and preparation of frozen sections can be achieved within five to ten minutes. The staining procedure used in this study takes an additional forty minutes. Photography and morphometric analysis can be completed in another ten to fifteen minutes. Therefore quantitative results can be produced by this technique approximately within an hour. It should be noted however that use of the software with confidence requires proper training.

The goal of the present study was to evaluate the suitability of our technique in determining the degree of ischemic injury. We therefore applied increasing ischemic intervals and evaluated the extent of injury with the software enhanced NADH-TR reaction. The results of our ischemic experiment showed that with our technique a continuous decrease in muscle viability could be demonstrated both in Type IIb and Type I fibers as well as in regard to total fiber viability. The obtained results indicated that Type I fibers suffered greater injury than Type IIb fibers. A strong correlation was discovered between muscle fiber viability and the length of ischemia in both examined fiber types, as well as in total fiber viability. Similar progressive decline in viability was previously observed using the planimetric method, however, without detection of such correlation with the length of ischemia [37]. Furthermore, in another study authors failed to detect any changes in viability during ischemia with the use of the planimetric method [42].

To verify that the obtained viability results represent true muscle injury, light- and electron microscopic evaluations were performed. No morphological changes were present after shorter periods of ischemia, a finding which was in consistency with previous data [43]. Electron microscopic results showed moderate degree of muscle injury after 8 hours of ischemia. After 9 hours, the degree of ultrastructural muscle damage increased considerably in all fiber types. Also, it was discovered that mitochondria-rich fibers (which most likely represent the oxidative fibers: such as Type IIa, or more likely Type I) sustained higher degree of ischemic damage than mitochondria-poor ones (supposedly Type IIb fibers). Therefore, the viability results obtained by our method correlate well with the electron microscopic findings in case of ischemic injury, since increasing ultrastructural damage resulted in a continuous decrease in muscle fiber viability, furthermore the differences between fiber types showed similar pattern by both electron microscopy and muscle fiber viability.

The fact that various fiber types respond differently to ischemia is well known [44]. There is some controversy regarding this topic, however to this date mainly Type IIb fibers have been reported to be more sensitive to ischemia [45]–[48]. By contrast, in our study we found that Type I fibers were the first to display the signs of injury and to show higher degree of injury than Type IIb fibers. This controversy might be resolved by assuming that complete ischemia – applied by the majority of the mentioned studies – (with no residual flow present; e.g. in case of tourniquet application) effects mostly the Type IIb, fast-twitch glycolytic fibers [49], whereas incomplete ischemia (such as aortic occlusion) might be more harmful to the Type I, slow-twitch oxidative fibers [15].

Reperfusion can paradoxically aggravate the degree of injury [50], [51], thus in the second part of our experiment we further examined the sensitivity of our method after revascularization.

In the 9IR group excessive muscle necrosis could be observed even in semithin sections, accompanied by almost complete cessation of viability. In the 8IR group muscle damage progression was also observable as compared with the 8I group. Both by electron microscopy and the viability test, approximately the same level of injury was detectable in the 8IR group as in the 9-hour-ischemia-only group. These findings support the feasibility of the assessment of muscle fiber viability, since similar morphological muscle damage resulted in similar viability results (Figure 5). Furthermore, the ultrastructural differences between fiber types in the 8IR group were also explored by the viability measurements.

Given that the results of total fiber viability were manifested as the median of the values obtained for the two separate fiber types and further that the results of the two fiber types did not differ significantly from the total fiber viability, the measurement of viability of separate fiber types is not required in order to assess the degree of injury. Assessment of total fiber viability is sufficient enough to produce reliable results.

It should be noted however that while our technique is faster than the other methods mentioned, and also that by using frozen samples the technique provides possibility for intra-operative diagnosis, the one hour sample processing time might not be fast enough. Since the staining procedure is the longest part of the sample processing period, reducing its time to a minimum might result in a clinically more relevant time frame. To achieve this however, further studies are required to specify the shortest sample processing time, which would provide the same specificity. Nevertheless, the utilization of frozen sections carries the possibility of simultaneous rapid routine histologic evaluation, which could significantly contribute to the correct therapeutic decision.

The lipid-like structures observable in places of mitochondria is a finding rarely addressed. These structures detected in our experiments are mainly inhomogeneous in structure, suggesting that these bodies are of mitochondrial origin rather than being actual storage lipid droplets. Our assumption is in accordance with previous results of others [52], [53]. It seems likely that the fine technique of perfusion fixation followed by osmium post-fixation can conserve these structures and increase their noticeability. The facts that these structures are present in large numbers only in the severely injured muscle fibers, and also that they are visible with light microscopy suggest that examination of semithin sections of perfusion fixed muscles may be sufficient to detect severe muscle injury, without need for further electron microscopic examination.

## Conclusion

In the current study authors present a novel diagnostic method, which might be suitable for the rapid determination of the degree of muscle injury. The results obtained by quantitative analysis showed a continuous decline in viability, which strongly correlated with the length of ischemia. Furthermore, the viability values represented the ultrastructural injury well, as seen on the electron micrographs; namely, high grade ultrastructural damage displayed low viability values and similar ultrastructural injury characteristics correlated to similar viability results. This technique might therefore have the potential to be translated into clinical practice in order to enable accurate therapeutic decisions regarding long-lasting lower limb acute arterial occlusions.
